# Quinoline-2-carbonitrile

**DOI:** 10.1107/S1600536810033118

**Published:** 2010-08-25

**Authors:** Wan-Sin Loh, Ching Kheng Quah, Madhukar Hemamalini, Hoong-Kun Fun

**Affiliations:** a X-ray Crystallography Unit, School of Physics, Universiti Sains Malaysia, 11800 USM, Penang, Malaysia

## Abstract

In the title compound, C_10_H_6_N_2_, the mol­ecule is almost planar, with an r.m.s. deviation of 0.014 Å. The dihedral angle between the aromatic rings is 1.28 (16)°. In the crystal, mol­ecules are stacked along the *a* axis by way of weak aromatic π–π stacking inter­actions between the benzene and pyridine rings of adjacent mol­ecules [centroid–centroid separation = 3.7943 (19) Å].

## Related literature

For the biological activity and syntheses of quinoline derivatives, see: Sasaki *et al.* (1998 ▶); Reux *et al.* (2009 ▶). For related structures, see: Fun *et al.* (2010 ▶); Loh *et al.* (2009 ▶, 2010 ▶). For the stability of the temperature controller used in the data collection, see: Cosier & Glazer (1986 ▶). For bond-length data, see: Allen *et al.* (1987 ▶).
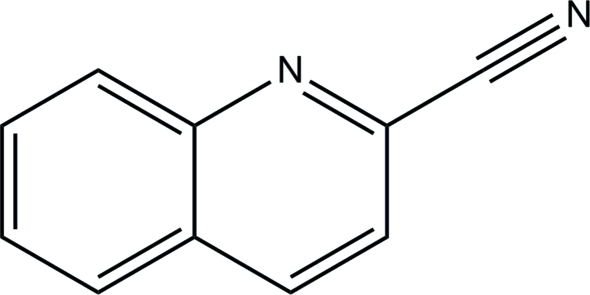


## Experimental

### 

#### Crystal data

C_10_H_6_N_2_
*M* *_r_* = 154.17
Orthorhombic, 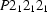

*a* = 3.8497 (2) Å
*b* = 9.9559 (4) Å
*c* = 19.9639 (13) Å
*V* = 765.16 (7) Å^3^
*Z* = 4
Mo *K*α radiation
μ = 0.08 mm^−1^
*T* = 100 K
0.36 × 0.18 × 0.03 mm

#### Data collection

Bruker SMART APEXII CCD diffractometer
Absorption correction: multi-scan (*SADABS*; Bruker, 2009 ▶) *T*_min_ = 0.971, *T*_max_ = 0.997
4086 measured reflections
1056 independent reflections
838 reflections with *I* > 2σ(*I*)
*R*_int_ = 0.059

#### Refinement

*R*[*F* ^2^ > 2σ(*F* ^2^)] = 0.056
*wR*(*F* ^2^) = 0.141
*S* = 1.09
1056 reflections
109 parameters
H-atom parameters constrained
Δρ_max_ = 0.31 e Å^−3^
Δρ_min_ = −0.24 e Å^−3^

### 

Data collection: *APEX2* (Bruker, 2009 ▶); cell refinement: *SAINT* (Bruker, 2009 ▶); data reduction: *SAINT*; program(s) used to solve structure: *SHELXTL* (Sheldrick, 2008 ▶); program(s) used to refine structure: *SHELXTL*; molecular graphics: *SHELXTL*; software used to prepare material for publication: *SHELXTL* and *PLATON* (Spek, 2009 ▶).

## Supplementary Material

Crystal structure: contains datablocks global, I. DOI: 10.1107/S1600536810033118/hb5597sup1.cif

Structure factors: contains datablocks I. DOI: 10.1107/S1600536810033118/hb5597Isup2.hkl

Additional supplementary materials:  crystallographic information; 3D view; checkCIF report

## Figures and Tables

**Fig. 1.**
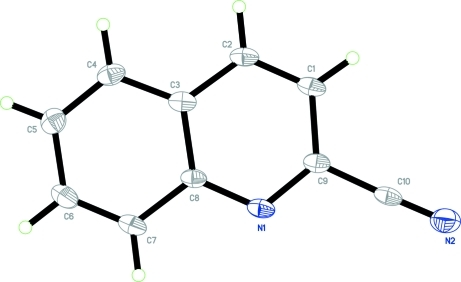
The molecular structure of (I) showing 50% probability displacement ellipsoids for non-H atoms.

**Fig. 2.**
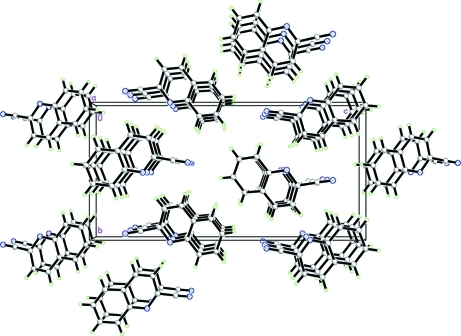
The crystal structure of (I), viewed along the a axis.

## supplementary crystallographic information

### Comment

Heterocyclic molecules containing cyano group are useful as drug intermediates. Syntheses of the quinoline derivatives were discussed earlier (Sasaki *et al.*, 1998; Reux *et al.*, 2009). Recently, we have synthesized a number of quinoline compounds to investigate the hydrogen bonding patterns in these compounds (Loh *et al.*, 2009; 2010). Herein we report the crystal structure of quinoline-2-carbonitrile.

In the title compound, Fig. 1, the molecule is almost planar with an r.m.s. deviation of 0.014 Å. The dihedral angle between the benzene (C3–C8) and pyridine (C1–C3/N1/C8/C9) rings is 1.28 (16)°. The bond lengths (Allen *et al.*, 1987) and angles in the title compound are within normal ranges and comparable to the related structure of quinoxaline-2-carbonitrile (Fun *et al.*, 2010).

In the crystal packing, Fig. 2, the molecules are stacked along the *a* axis by way of weak aromatic π–π stacking interactions between the centroid of the benzene (*Cg*1) and the centroid of the pyridine (*Cg*2) rings of adjacent molecules [*Cg*1···*Cg*2 separation = 3.7943 (19) Å]. There is no significant hydrogen bond observed in this compound.

### Experimental

A hot methanol solution (20 ml) of quinoline-2-carbonitrile (39 mg, Aldrich) was warmed over a magnetic stirrer hotplate for a few minutes. The resulting solution was allowed to cool slowly to room temperature. Colourless plates of (I) appeared after a few days.

### Refinement

All H atoms were positioned geometrically with the bond length of C–H being 0.93 Å and were refined using a riding model, with *U*_iso_(H) = 1.2 *U*_eq_(C). In the absence of significant anomalous dispersion, 616 Friedel pairs were merged for the final refinement.

### Crystal data

**Table:**

C_10_H_6_N_2_	*F*(000) = 320
*M**_r_* = 154.17	*D*_x_ = 1.338 Mg m^−^^3^
Orthorhombic, *P*2_1_2_1_2_1_	Mo *K*α radiation, λ = 0.71073 Å
Hall symbol: P 2ac 2ab	Cell parameters from 930 reflections
*a* = 3.8497 (2) Å	θ = 3.7–27.4°
*b* = 9.9559 (4) Å	µ = 0.08 mm^−^^1^
*c* = 19.9639 (13) Å	*T* = 100 K
*V* = 765.16 (7) Å^3^	Plate, colourless
*Z* = 4	0.36 × 0.18 × 0.03 mm

### Data collection

**Table:**

Bruker SMART APEXII CCD diffractometer	1056 independent reflections
Radiation source: fine-focus sealed tube	838 reflections with *I* > 2σ(*I*)
graphite	*R*_int_ = 0.059
φ and ω scans	θ_max_ = 27.5°, θ_min_ = 3.7°
Absorption correction: multi-scan (*SADABS*; Bruker, 2009)	*h* = −4→4
*T*_min_ = 0.971, *T*_max_ = 0.997	*k* = −12→12
4086 measured reflections	*l* = −25→21

### Refinement

**Table:**

Refinement on *F*^2^	Primary atom site location: structure-invariant direct methods
Least-squares matrix: full	Secondary atom site location: difference Fourier map
*R*[*F*^2^ > 2σ(*F*^2^)] = 0.056	Hydrogen site location: inferred from neighbouring sites
*wR*(*F*^2^) = 0.141	H-atom parameters constrained
*S* = 1.09	*w* = 1/[σ^2^(*F*_o_^2^) + (0.0827*P*)^2^] where *P* = (*F*_o_^2^ + 2*F*_c_^2^)/3
1056 reflections	(Δ/σ)_max_ < 0.001
109 parameters	Δρ_max_ = 0.31 e Å^−^^3^
0 restraints	Δρ_min_ = −0.24 e Å^−^^3^

### Special details

**Table:**

Experimental. The crystal was placed in the cold stream of an Oxford Cryosystems Cobra open-flow nitrogen cryostat (Cosier & Glazer, 1986) operating at 100.0 (1) K.
Geometry. All e.s.d.'s (except the e.s.d. in the dihedral angle between two l.s. planes) are estimated using the full covariance matrix. The cell e.s.d.'s are taken into account individually in the estimation of e.s.d.'s in distances, angles and torsion angles; correlations between e.s.d.'s in cell parameters are only used when they are defined by crystal symmetry. An approximate (isotropic) treatment of cell e.s.d.'s is used for estimating e.s.d.'s involving l.s. planes.
Refinement. Refinement of *F*^2^ against ALL reflections. The weighted *R*-factor *wR* and goodness of fit *S* are based on *F*^2^, conventional *R*-factors *R* are based on *F*, with *F* set to zero for negative *F*^2^. The threshold expression of *F*^2^ > σ(*F*^2^) is used only for calculating *R*-factors(gt) *etc*. and is not relevant to the choice of reflections for refinement. *R*-factors based on *F*^2^ are statistically about twice as large as those based on *F*, and *R*- factors based on ALL data will be even larger.

### Fractional atomic coordinates and isotropic or equivalent isotropic displacement parameters (Å^2^)

**Table:**

	*x*	*y*	*z*	*U*_iso_*/*U*_eq_	
N1	0.7386 (8)	0.5083 (2)	0.20498 (14)	0.0185 (6)	
N2	0.3930 (9)	0.4374 (2)	0.35822 (16)	0.0296 (7)	
C1	0.7983 (9)	0.2683 (3)	0.22614 (16)	0.0193 (7)	
H1A	0.7552	0.1964	0.2547	0.023*	
C2	0.9617 (9)	0.2502 (3)	0.16647 (16)	0.0192 (7)	
H2A	1.0368	0.1649	0.1541	0.023*	
C3	1.0178 (9)	0.3604 (3)	0.12314 (16)	0.0174 (7)	
C4	1.1815 (8)	0.3487 (3)	0.06064 (16)	0.0201 (7)	
H4A	1.2662	0.2658	0.0468	0.024*	
C5	1.2177 (9)	0.4589 (3)	0.01965 (18)	0.0224 (7)	
H5A	1.3204	0.4498	−0.0223	0.027*	
C6	1.0989 (8)	0.5855 (3)	0.04129 (17)	0.0239 (8)	
H6A	1.1257	0.6595	0.0133	0.029*	
C7	0.9466 (9)	0.6018 (3)	0.10188 (17)	0.0213 (7)	
H7A	0.8736	0.6866	0.1154	0.026*	
C8	0.8981 (8)	0.4894 (3)	0.14492 (17)	0.0162 (7)	
C9	0.6962 (8)	0.4002 (3)	0.24322 (16)	0.0169 (7)	
C10	0.5263 (9)	0.4229 (3)	0.30714 (17)	0.0196 (7)	

### Atomic displacement parameters (Å^2^)

**Table:**

	*U*^11^	*U*^22^	*U*^33^	*U*^12^	*U*^13^	*U*^23^
N1	0.0149 (13)	0.0120 (10)	0.0284 (15)	−0.0014 (10)	−0.0013 (13)	−0.0012 (10)
N2	0.0336 (17)	0.0199 (13)	0.0353 (17)	0.0000 (12)	0.0055 (16)	−0.0013 (13)
C1	0.0146 (16)	0.0128 (12)	0.0306 (18)	0.0016 (12)	−0.0030 (15)	0.0009 (12)
C2	0.0155 (16)	0.0113 (12)	0.0307 (18)	0.0009 (12)	−0.0020 (15)	−0.0027 (12)
C3	0.0130 (15)	0.0150 (13)	0.0243 (17)	0.0006 (11)	−0.0063 (14)	−0.0028 (12)
C4	0.0134 (16)	0.0184 (14)	0.0286 (19)	0.0016 (12)	−0.0010 (15)	−0.0060 (13)
C5	0.0164 (16)	0.0251 (15)	0.0256 (17)	−0.0004 (14)	−0.0003 (15)	−0.0006 (13)
C6	0.0218 (17)	0.0167 (13)	0.0333 (19)	−0.0009 (13)	−0.0003 (16)	0.0053 (14)
C7	0.0202 (17)	0.0128 (13)	0.0311 (19)	−0.0003 (13)	−0.0039 (16)	0.0021 (12)
C8	0.0124 (15)	0.0111 (12)	0.0249 (16)	−0.0016 (11)	−0.0027 (14)	−0.0033 (12)
C9	0.0130 (14)	0.0130 (12)	0.0247 (17)	−0.0019 (12)	−0.0035 (14)	−0.0018 (11)
C10	0.0186 (17)	0.0098 (12)	0.0306 (19)	−0.0019 (12)	−0.0015 (15)	0.0008 (12)

### Geometric parameters (Å, °)

**Table:**

N1—C9	1.329 (4)	C4—C5	1.376 (4)
N1—C8	1.360 (4)	C4—H4A	0.9300
N2—C10	1.151 (4)	C5—C6	1.409 (4)
C1—C2	1.359 (4)	C5—H5A	0.9300
C1—C9	1.413 (4)	C6—C7	1.354 (4)
C1—H1A	0.9300	C6—H6A	0.9300
C2—C3	1.414 (4)	C7—C8	1.424 (4)
C2—H2A	0.9300	C7—H7A	0.9300
C3—C4	1.402 (4)	C9—C10	1.452 (4)
C3—C8	1.432 (4)		
			
C9—N1—C8	116.7 (2)	C6—C5—H5A	120.1
C2—C1—C9	117.6 (3)	C7—C6—C5	121.5 (3)
C2—C1—H1A	121.2	C7—C6—H6A	119.3
C9—C1—H1A	121.2	C5—C6—H6A	119.3
C1—C2—C3	120.3 (3)	C6—C7—C8	120.1 (3)
C1—C2—H2A	119.9	C6—C7—H7A	120.0
C3—C2—H2A	119.9	C8—C7—H7A	120.0
C4—C3—C2	123.3 (2)	N1—C8—C7	118.8 (2)
C4—C3—C8	119.3 (3)	N1—C8—C3	122.5 (3)
C2—C3—C8	117.4 (3)	C7—C8—C3	118.7 (3)
C5—C4—C3	120.6 (3)	N1—C9—C1	125.4 (3)
C5—C4—H4A	119.7	N1—C9—C10	115.7 (2)
C3—C4—H4A	119.7	C1—C9—C10	118.8 (3)
C4—C5—C6	119.9 (3)	N2—C10—C9	178.2 (3)
C4—C5—H5A	120.1		
			
C9—C1—C2—C3	−1.4 (4)	C6—C7—C8—N1	−178.8 (3)
C1—C2—C3—C4	−179.4 (3)	C6—C7—C8—C3	0.9 (5)
C1—C2—C3—C8	0.3 (4)	C4—C3—C8—N1	−179.7 (3)
C2—C3—C4—C5	177.7 (3)	C2—C3—C8—N1	0.6 (4)
C8—C3—C4—C5	−2.0 (4)	C4—C3—C8—C7	0.6 (4)
C3—C4—C5—C6	1.9 (5)	C2—C3—C8—C7	−179.1 (3)
C4—C5—C6—C7	−0.4 (5)	C8—N1—C9—C1	−1.0 (5)
C5—C6—C7—C8	−1.0 (5)	C8—N1—C9—C10	179.8 (3)
C9—N1—C8—C7	179.4 (3)	C2—C1—C9—N1	1.8 (5)
C9—N1—C8—C3	−0.3 (4)	C2—C1—C9—C10	−179.0 (3)
